# Eosinophilic disorders: evaluation of current classification and diagnostic criteria, proposal of a practical diagnostic algorithm

**DOI:** 10.1186/s13601-019-0277-4

**Published:** 2019-07-25

**Authors:** Polliana Mihaela Leru

**Affiliations:** 1 Carol Davila University of Medicine and Pharmacy, Bucharest, Romania; 2 Internal Medicine Department, Colentina Clinical Hospital, Sos. Stefan cel Mare, No. 19-21, District 2, 020125 Bucharest, Romania

**Keywords:** Diagnostic criteria, Eosinophilic disorders, Hypereosinophilia, Hypereosinophilic syndrome, Practical diagnostic algorithm

## Abstract

Eosinophilic disorders represent a group of pathologic conditions with highly heterogeneous pathophysiology and clinical presentation and variable prognosis, ranging from asymptomatic or mild, to severe and complex cases, with fatal outcome. Interest in this group of disorders has increased during the last two decades, with consistent progress made regarding understanding of molecular mechanisms, refining of diagnostic criteria, classification and evaluation of therapeutic options. There are still many gaps and difficulties in evaluating eosinophilic syndromes and diseases in medical practice. The disease prognosis depends mainly on the cause and mechanism of eosinophilia, on severity of organ dysfunction and on accurate diagnosis and response to treatment. Besides primary hypereosinophilic syndromes and secondary (reactive) eosinophilias, many associated or idiopathic forms have been described, making this topic a complex and difficult medical entity. An important aim of the experts in the field is to agree upon a more clear and practically useful classification, a better characterization of various phenotypes and endotypes of eosinophilic diseases and to identify novel biomarkers and more effective therapies. The aim of this paper is to review recent data from the literature regarding definition, classification and diagnosis criteria of eosinophilic diseases and to propose a revised and updated diagnostic algorithm useful in clinical practice.

## Introduction

Eosinophilic diseases represent a broad range of pathologic conditions characterized by various degree of persistent blood and/or tissue hypereosinophilia, with potential for end-organ dysfunction [1]. Interest in this group of disorders has recently increased with consistent progress made regarding understanding of molecular mechanisms, refining of diagnostic criteria, classification and evaluation of therapeutic options. The pathophysiology and clinical presentation of eosinophilic disorders are highly heterogeneous and disease outcome may vary from asymptomatic or mild, to severe and fatal, with variable time course patterns. There are significant geographical influences regarding the most prevalent causes of hypereosinophilia, with reported parasitic infectious in tropical settings and allergic diseases in more developed and occidental countries. Many other hematologic (primary) and non-hematologic (reactive or secondary) hypereosinophilic syndromes and diseases, as well as associated or idiopathic forms have been described, making this topic a complex and difficult medical entity to unravel. There are still many gaps and difficulties in evaluating eosinophilic syndromes and diseases in medical practice, despite recommendations from consensus papers regarding diagnostic criteria and management. An important aim of the experts in the field is agreement upon diagnosis criteria and better characterization of various phenotypes, a clearer and more practically useful classification, with a focus on distinguishing isolated or asymptomatic eosinophilias from diseases with specific and potentially severe eosinophil-related organ damage, identification of novel biomarkers and more effective therapies [2]. Another important aspect in medical practice is the need for multidisciplinary and personalized approach of the patient with hypereosinophilia and evaluation of disease burden for patients and health systems.

## Definition and epidemiology of eosinophilic disorders

The upper limit of the absolute eosinophil count (AEC) in the peripheral blood is considered between 350 and 500/mm^3^ and a percentage of 3–5% of the total white blood cell count. The term eosinophilia is recommended for a small increase of AEC from the upper limit to 1500/mm^3^. Hypereosinophilia (HE) is defined based on an AEC greater than 1500/mm^3^ on two consecutive occasions, persistent for a minimum of 1 month (instead of 6 months, as previously considered in the definition of hypereosinophilic syndrome (HES) [1]. According to severity-based classification of hypereosinophilia, HE is considered moderate in case of AEC between 1500 and 5000/mm^3^ and severe, if AEC is more than 5000/mm^3^. The 2010 revision of HES diagnosis criteria recommended that a peripheral AEC > 1500/mm^3^ not be a requirement for HES diagnosis, as previously considered, due to possible discrepancies between blood and tissue eosinophilia [3]. Eosinophilic disorders are defined by organ dysfunction induced by activated eosinophils and this can be single-organ disease or multiple-organ disease, accompanied by variable degree of blood eosinophilia. The same revision of diagnostic criteria recommended keeping the provisional diagnosis of idiopathic eosinophilic disorder for the subgroup of patients who have blood eosinophilia > 1500/mm^3^, but without end-organ dysfunction, since they may benefit from regular monitoring. From etiopathogenic point of view, eosinophilic disorders are classified as secondary (or reactive) to a broad range of causal factors, such as infections and allergens, or primary, when no causal factor can be identified. Other terms have been used in the previous classifications, to define less well characterized eosinophilic syndromes, such as idiopathic, associated, overlap or HE with uncertain or undetermined significance.

Epidemiology of eosinophilic disorders is not clear, mainly due to variable clinical picture and involvement of many specialists, but also due to still inconsistent definition and diagnosis criteria. There are no data regarding incidence and prevalence of all types of eosinophilic disorders, which should be probably evaluated based on geographic regional approach. The reported age-adjusted incidence rate of HES, including chronic eosinophilic leukemia, based on the International Classification of Disease (ICD) for Oncology (version 3), was about 0.036 per 100.000, as resulting from the Surveillance, Epidemiology and End Results (SEER) database between 2001 and 2005 [4]. A study evaluating the spectrum of eosinophilia in a tropical setting reported the incidence of 0.5 to 1 case/100.000 hospital population for HE/HES, which is likely to be underestimated [5].

## The concept of hypereosinophilic syndrome

The initial concept of hypereosinophilic syndrome (HES) was introduced by Hardy and Anderson in 1968, corresponding to a severe clinical entity defined by persistent blood eosinophilia without clear cause, multiorgan involvement and fatal outcome [6]. The first diagnostic criteria of primary or idiopathic HES were stated in 1975 by Chusid, including the following: persistent blood absolute eosinophil count over 1500/mm^3^ for a duration of more than 6 months, with evidence of tissue and organ damage, without any identifiable cause of eosinophilia [7]. It was clear from that time that a subgroup of patients with HES had clinical and hematological characteristics of myeloproliferative disease, such as splenomegaly, anemia and myelofibrosis. The identification, in 2003, of the tyrosine kinase fusion gene mutation, associated with this subgroup, allowed better characterization and a specific targeted treatment with tyrosine kinase inhibitors [8]. Since the early 2000s, the definition, terminology and diagnostic criteria of different subgroups of hypereosinophilic syndromes and diseases have been revised by many panels of experts, mainly hematologists. One classification of myeloid neoplasms, including primary HES was proposed by World Health Organization (WHO) in 2008 and was later revised in 2016 [9]. A more complex classification of eosinophilic disorders was proposed in 2011 by a larger international and multidisciplinary panel of experts, called the International Working Group on Eosinophil Disorders (ICOG-EO), who agreed on terminology and diagnostic criteria of various forms of HE and HES [10]. This classification retained the criteria referring to the level of blood eosinophilia, but changed the qualifying duration from 6 to 1 month, adding tissue eosinophilia and including some particular conditions, such as asymptomatic, associated and overlap forms of eosinophilia. A more recently published paper included a critical appraisal of the current classification of HES and proposed a clearer and more practical definition of various types and subgroups of HE and HES [2] (Table 1). Inconsistent correlation between severity of blood hypereosinophilia and clinical picture of eosinophilic disorders has been reported in the literature, since tissue eosinophilia may persist and induce organ dysfunction despite lowering the blood eosinophilia. Recent research suggests that serum eosinophil cationic protein (ECP) may prove to be a useful serological marker of eosinophil activation, which may account for these discrepancies, but further research for confirmation of diagnostic value of ECP in eosinophilic disorders is needed [11].

**Table 1:** Summary of ICOG definitions and diagnosis criteria of eosinophilic disorders (adapted from Kahn et al. [2])

Terminology	Definition and criteria
Blood eosinophilia	Eosinophils > 0.5 × 10^9^/L
Hypereosinophilia (HE)	Eosinophils > 1.5 × 10^9^/L in blood on 2 examinations (interval > 1 month) and/or tissue HE defined by the following: 1. Percentage of eosinophils in bone marrow section exceeds 20% of all nucleated cells and/or 2. Extensive tissue infiltration by eosinophils based on pathologist report and/or 3. Marked deposition of eosinophil granule proteins (in the absence or presence of major tissue infiltration by eosinophils)
Secondary (reactive) HE	Clinical and laboratory evidence for causes of HE: 1. Common allergic, reactive or immunologic conditions 2. Hematopoietic neoplasms 3. Non-hematopoietic neoplasms (paraneoplastic HE) 4. Rare conditions associated with HE
Hypereosinophilic syndrome (HES)	1. Criteria for peripheral blood HE fulfilled and 2. Organ damage and/or dysfunction attributable to tissue HE, and 3. Exclusion of secondary (reactive) HE as major reason for organ damage
HE of undetermined significance	1. Criteria for peripheral blood HE fulfilled and 2. No clinical symptoms and/or proof of organ dysfunction
Overlap HE syndromes	Criteria for HES and EGPA (ANCA-negative subtype)
Associated HE disorders	1. Criteria of HE fulfilled and 2. Single-organ disease or Secondary (reactive) HE

*ANCA* antineutrophil cytoplasm antibody, *EGPA* eosinophil granulomatosis with polyangiitis

### Underlining mechanisms of hypereosinophilia and eosinophils activation

The key cytokines that are critical for stimulation of bone marrow production of eosinophils include interleukins IL-3, IL-5 and granulocyte/macrophage colony-stimulating factor (GM-CSF). These three cytokines are produced by T cells CD4 and CD8 from peripheral blood and inflammed tissues.IL-5 is the key cytokine in terminal differentiation of eosinophils and a major therapeutic target for new medications to treat hypereosinophilic conditions. Activated eosinophils have complex proinflammatory effects: direct cytotoxic against tissues and microorganisms, promote thrombosis, fibrosis and angiogenesis, tissue remodeling, platelet and endothelial cells activation. The degree and pattern of organ involvement in eosinophilic disorders are governed by two distinct factors: the increased production and/or persistent accumulation of normal or neoplastic eosinophils and persistent activation of eosinophils, which is responsible for the clinical manifestations of HES. The two principal pathogenic mechanisms that can trigger eosinophil growth and accumulation are either an intrinsic defect of eosinophil-committed neoplastic progenitor cells, caused by mutations including PDGFR or FGFR1 or cytokines overproduction such as IL-3 and IL-5, that stimulate the growth, differentiation and survival of eosinophils.

#### Reactive (secondary eosinophilia)

Eosinophilia can be secondary (reactive) to a large spectrum of causes, including infections, allergies, autoimmune and neoplastic disorders (Table 2). The most frequent causes of eosinophilia in developing and tropical countries are parasitic infections, particularly with tissue-invasive parasites, such as Toxocara species, Toxoplasma gondii, Strongyloides, Trichinella, Echinococcus, Microfilaria.

**Table 2:** Pathologic conditions associated with reactive hypereosinophilia [13]

Allergic atopic or non-atopic diseases	Eosinophilic asthma, allergic rhinitis, non-allergic rhinitis with eosinophilia syndrome (NARES), food allergies, atopic dermatitis, drug allergies (ex.DRESS), allergic bronchopulmonary aspergillosis (ABPA), eosinophilic chronic rhinosinusitis (ECRS), eosinophilic otitis media, eosinophilic laryngitis
Infections	Parasitic (Toxocara, Toxoplasma, Strongyloides, Ascariasis, Trichinella, Echinococcus, Scabiae, Microfilaria) Fungal (Coccidioides mycoses) Viral (HIV, HCV)
Autoimmune diseases	Connective tissue disorders, sarcoidosis, inflammatory bowel disease, bullous pemphigoid, systemic vasculitis (Wegener disease, Churg–Strauss syndrome)
Endocrine diseases	Addison’s disease
Hematologic neoplasms	Myeloid: acute/chronic eosinophilic leukemia, chronic myeloid leukemia Ph+, myelodysplastic syndromes, systemic mastocytosis, aggressive mastocytosis, mast cell leukemia Lymphoid: Hodgkin lymphoma, non-Hodgkin lymphoma, T-cell lymphoma
Solid neoplasms	Adenocarcinoma of the lung, gastro-intestinal tract, pancreas, thyroid, genital and skin tumors
Organ restricted diseases with HE	Esophagitis, gastroenteritis, cystitis, pneumonia, dermatologic conditions
Immunodeficiencies	Hyper IgE syndrome (Job’s syndrome) Omenn syndrome
Rare diseases	Gleich syndrome (episodic angioedema, eosinophilia, policlonal IgM) Eosinophilia-Mialgia syndrome
Other	Graft-versus-host disease, Cholesterol embolization, radiation exposure

In occidental European and other developed countries, eosinophilia may be reactive to various allergic diseases, such as respiratory allergies: eosinophilic asthma, rhinitis, chronic rhinosinusitis (ECRS), otitis media, laryngitis and atopic dermatitis, drug and food allergies, which have all an increasing prevalence during the last decades. The most severe forms of hypereosinophilia due to hypersensitivity reactions are allergic broncho-pulmonary aspergillosis (ABPA) and delayed type of drug allergies, known as drug reaction with eosinophilia and systemic symptoms (DRESS). Other causes of eosinophilia are autoimmune and inflammatory disorders, such as: systemic lupus erythematosus, eosinophilic granulomatosis with polyangiitis known as Churg–Strauss syndrome (EGPA), Wegener’s disease, pulmonary eosinophilic disorders, adrenal insufficiency. In non-myeloid hematologic and solid neoplasms, eosinophilia results from the production of cytokines by malignant cells, mainly interleukin (IL)-5, such as in T cell and Hodgkin lymphoma, acute lymphoblastic leukemia. Eosinophilic disorders of the gastrointestinal tract, mainly eosinophilic esophagitis, gastroenteritis and colitis, may be accompanied by peripheral eosinophilia in a significant number of patients and this is considered an independent predictor of relapsing disease. It was reported that progressive blood eosinophilia associated with multiple food allergies may precede onset of gastrointestinal symptoms with months-years, confirming the importance of early allergist evaluation in the diagnosis work-up of hypereosinophilia [12].

#### Primary hypereosinophilic syndromes

Eosinophilic disorders may be considered as primary hypereosinophilic syndromes after comprehensive evaluation and exclusion of secondary causes of eosinophilia, based on some defined characteristics. In recent classifications, HES has been divided in multiple subgroups, based on clinical, laboratory and molecular features. The two major categories of HES are myeloproliferative HES (M-HES) and lymphocytic HES (L-HES), with some other overlap, associated or less-well defined clinical entities, also included in this large group (Table 3). All these described subtypes of HES taken together represent about 50% of HES cases, meaning that at present almost half of the cases do not meet diagnostic criteria for any of the defined subtypes, thus requiring future characterization. The identification of the mechanism and molecular characterization of HES subtype is essential for predicting clinical outcome, treatment selection and prognosis.

**Table 3:** The main subtypes of primary (neoplastic) hypereosinophilic syndromes [13]

Subtype	Clinical features	Laboratory tests
Myeloproliferative (M-HES)	Male predominance Hepato-splenomegaly Anemia Endomyocardial fibrosis Restrictive lung disease Mucosal ulcerations Good response to imatinib Variable steroid response Poor prognosis	F/P gene mutation by RT-PCR or FISH Increased serum tryptase Increased serum B12 Thrombocytopenia Dysplastic eosinophils Myelofibrosis Myeloid precursors in blood
Lymphocytic (L-HES)	Almost equal sex ratio History of atopy Frequent skin lesions Gastrointestinal symptoms Obstructive lung disease Low mortality Possible progression to T-cell lymphoma Rare cardiac involvement Steroid responsive	Aberrant phenotypic T-cell population in blood Clonal T-cell pattern by PCR Increased eosinophilopoietic cytokines (IL-5) Increased serum IgE Increased TARC

*TARC* thymus activation-regulated chemokine, *F/P mutation* FIP1L1-PDGFRA mutation, *FISH* fluorescence in situ hybridization, *RT-PCR* reverse transcriptase polymerase chain reaction

1. *Myeloproliferative variant of HES* is a severe form of HES, including either a defined form of myeloid malignancy or some blood and bone marrow abnormalities characteristic for myeloproliferative disorders, associated with eosinophilia and end-organ damage, due to infiltration with activated eosinophils. The main clinical features of M-HES are: male predominance, hepato-splenomegaly, anemia, thrombocytopenia, severe organ involvement, and poor prognosis, corresponding to the initial description of HES in early 1970s [7]. The molecular defect responsible for this distinct phenotype is a gene fusion of FIP1-like 1 (FIPL1) and platelet-derived growth factor receptor alpha (PDGFRA) (F/P), leading to a gain of function, due to a constitutively active tyrosine kinase. The result is a persistent production and expansion of eosinophils from hematopoietic stem cells. This mutation can be detected by FISH or RT-PCR of peripheral blood or bone marrow and has very important therapeutic consequences, as the therapeutic target for imatinib—an inhibitor of tyrosine kinase [14]. The reported incidence of F/P mutation in HES patients was 11% [15]. Some of the M-HES patients may have clinical and laboratory characteristics of the myeloproliferative subtype, but negative F/P mutation or may have other mutations, such as PDGFRB or FGFR1 [16]. These mutations may also lead to clonal eosinophlia, with potential progression to myeloid malignancies and possibly refractory to treatment. Diagnosis of M-HES with negative F/P mutation may be confirmed based on at least four of the following criteria: dysplastic eosinophils in blood, high serum level of tryptase and vitamin B12, anemia and/or thrombocytopenia, hepato-splenomegaly, bone marrow cellularity > 80%, myelofibrosis or spindle-shaped mast cell [17]. Myeloproliferative neoplasms (MPN) and myelodysplastic syndromes (MDS) associated with hypereosinophilia include a large list of myeloid neoplasms, according to WHO revised classification from 2008 and reaffirmed in 2016 [18]. A subset of patients with M-HES may have elevated serum tryptase, mast cell infiltration of the bone marrow and poor prognosis [19]. When F/P screening is not available, evaluation of the serum tryptase can be a useful surrogate marker for F/P positive disease, since increased levels segregate with this molecular abnormality.
2. *Lymphocytic*—*variant of HES*
  Lymphocytic variant of HES is a less clearly defined diagnosis entity, with hypereosinophilia due to overproduction of eosinophilopoietic cytokines, by a clonal population of activated T-lymphocytes (T-cells). The mechanism can be considered both clonal and reactive, since eosinophilia is reactive to the eosinophilopoietic growth factors (mainly IL-5, but also IL-4 and IL-13) produced by the abnormal population of T-lymphocytes, with atypical pattern of surface markers and a T helper cell type 2 (Th2) cytokine profile [20]. From HES current classification perspective, the term reactive, used in this case, may induce confusion with secondary eosinophilia. The abnormal T-cells from peripheral blood can be characterized by flow cytometry and/or cell receptor rearrangement studies. In some patients from the L-HES subgroup, the clonal T-cell populations may not be identified, but aberrant immunophenotyping and evidence of activated T-cells may provide additional support for diagnosing L-HES. The clinical pattern of L-HES is characterized by cutaneous signs, such as urticaria and symptoms due to other organs involvement, such as adenomegaly, rheumatologic, gastrointestinal, pulmonary, neurologic and cardio-vascular diseases [21]. A possible disease progression to lymphoma was mentioned in the literature [22]. The clinical course may be longer, but tissue and marrow fibrosis is uncommon. The prevalence of lymphocytic variant is estimated at 10–15% of HES, but many cases can not be confirmed and may be included in other categories of HES, due to variable clinical picture and to incomplete diagnosis criteria.
  *Other categories of HES* defined based on clinical pattern are: familial, episodic, overlap, associated, idiopathic and hypereosinophilia with undetermined (uncertain) significance.
3. *Familial hypereosinophilia* is a rare condition, characterized by blood eosinophilia with unclear cause, repeated in successive generations. The affected members of the family may be asymptomatic or may develop severe clinical manifestations similar to HES with positive F/P mutation, such as cardiac fibrosis and neurologic abnormalities [23]. The mechanism of hypereosinophilia appears a mutation of the gene located on chromosome 5q31-33, the cytokine gene cluster, the same region as genes crucial for eosinophilia—IL-5, IL-3 and GM-CSF.
4. *Episodic hypereosinophilia* is known as Gleich’ syndrome or episodic angioedema with eosinophilia (EAE), a rare disease characterized by recurrent angioedema accompanied by hypereosinophilia, with cyclical variable clinical pattern. It is considered a form of L-HES variant, due to described aberrant populations of T-lymphocytes and the cytokine profile [24].
5. *Hypereosinophilia of undetermined significance* is rather a provisional diagnosis, until extensive evaluation of a HES or a benign form of eosinophilic disease, with long time persistent blood eosinophilia more than 1500/mm^3^, but no signs of end-organ dysfunction attributable to eosinophilia [25]. According to a large experts consensus, these patients require close monitoring, since they may develop eosinophilic end-organ disease and clinical symptoms at any time of persistent peripheral eosinophilia [10]. No clear recommendations regarding clinical and laboratory parameters for monitoring are mentioned in current guidelines.
6. *Overlap eosinophilic disorders* are conditions that associate single organ-restricted eosinophilia, which may be preceded or accompanied by peripheral eosinophilia, such as eosinophilic gastrointestinal disorders (EGID), eosinophilic esophagitis, eosinophilic pneumonia, eosinophilia-myalgia syndrome [17].This category also includes the association between HES and eosinophilic granulomatosis with polyangiitis (EGPA) or Churg–Strauss syndrome, more precisely a subgroup of ANCA-negative patients with eosinophilic asthma, less frequent vasculitic manifestations (such as nephropathy, purpura, peripheral neuropathy, scleritis), but more frequent cardiomyopathy, mimicking M-HES [26]. Due to the clinical and biological profile of these patients and also to the good response to mepolizumab, it was suggested to diagnose this entity as a subtype of HES, named hypereosinophilic asthma with systemic (non-vasculitic) manifestations (HASM), rather than EGPA [27].
7. *Associated eosinophilic disorders* include various subtypes of HES associated with other conditions, known as causes of reactive HE, such as systemic mastocytosis, infections, inflammatory bowel disease, systemic vasculitis, other autoimmune diseases.
8. *Rare syndromes accompanied by HE* are inherited immunodeficiencies, such as Omenn syndrome and Hyper IgE (Job’s syndrome), usually manifested in children.
9. *Diagnosis of Idiopathic HES* requires exclusion of all primary and secondary causes of hypereosinophilia and may be maintained, based on incomplete diagnosis criteria, despite exhaustive evaluation of the patient with HE.

**Fig. 1:**
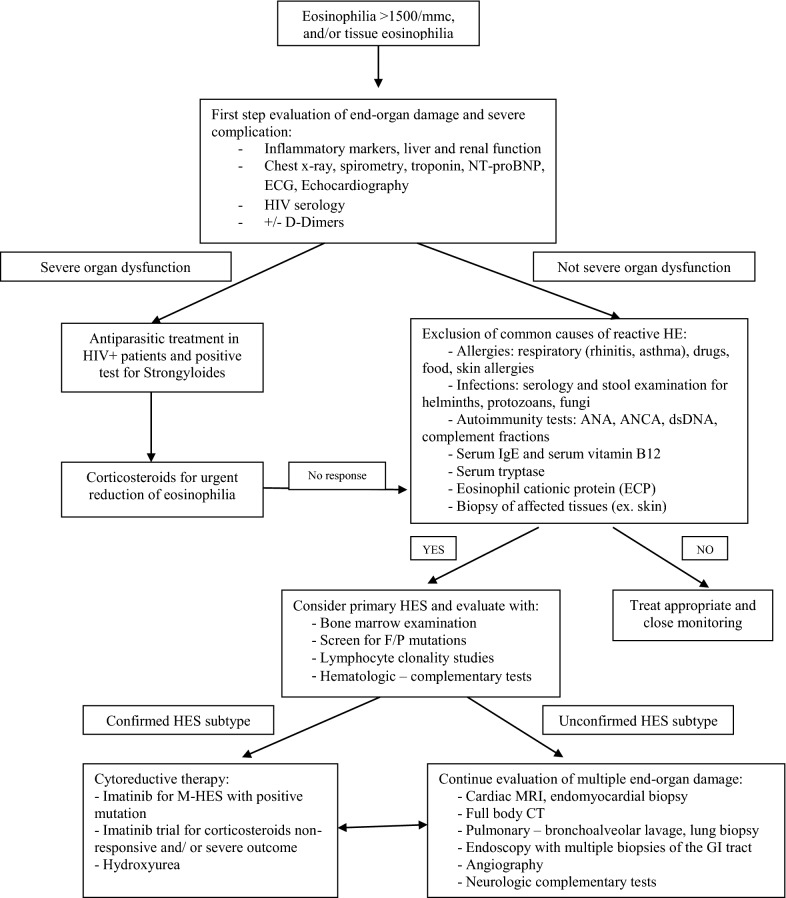
A proposed diagnostic algorithm of Hypereosinophilia and Eosinophilic diseases

Taking into consideration a step by step approach of hypereosinophilia in clinical practice and the main laboratory tests used for a comprehensive evaluation, a proposal of a diagnosis algorithm of HES is showed in Fig. 1.

## Complications and prognosis

Hypereosinophilia is associated with a wide spectrum of end-organ dysfunction and many possible complications. Despite initial benign clinical appearance in many cases, eosinophilia may sometimes have rapid and severe progression, with life-threatening prognosis [28]. Primary hypereosinophilic syndromes are malignant diseases with variable outcome, with prognosis depending on prompt evaluation and diagnosis confirmation, early complications and on the initial therapeutic response. The factors predictive of worse outcomes of HES are: male sex, the degree of eosinophilia, early cardiac disease, lack of response to corticosteroids and association of a myeloproliferative syndrome [29]. Cardiovascular complications of HES represent the worst prognosis, responsible for a high rate of fatal outcome. Very severe cases with fatal outcome due to myocardial necrosis and thromboembolic complications in less than 1 month after clinical onset of eosinophilic cardiac disease have been described in the literature [30, 31]. Thromboembolic events are major complications of eosinophilic disorders, with clinical manifestations depending on the affected territory. Possible neurologic complications are ischemic attacks, peripheral neuropathies and encephalopathy. Other possible complications of HES or associated forms of eosinophilic disorders are respiratory disease, which may appear in about 40% of cases. The respiratory clinical manifestations may be: severe asthma, infiltrative pulmonary disease, pleural effusions, pulmonary embolism or fibrosis [32]. Irrespective of HES subtype, some relevant clinical and laboratory indicators of poor prognosis may be considered: leukocytosis, very high and rapid progressive eosinophilia, bone marrow precursors in the blood and early cardiac involvement [33].

### Difficulties in evaluating eosinophilic disorders

The evaluation of a patient with eosinophilia may be complex, costly, needs time and a multidisciplinary approach. The involvement of allergist, hematologist, pathologist and the infectious diseases specialist may be recommended in diagnosis and management of eosinophilic disorders.

There are many difficulties in evaluation of eosinophilic syndromes and diseases in clinical practice, due to very heterogeneous clinical picture and multiple molecular and laboratory markers. The degree of HE may be orientative for etiology and for potentially severe disease outcome. While eosinophilia and moderate HE are usually encountered in allergic diseases and infections, very high HE may indicate more severe diseases, such as malignant HES. The hematological evaluation of HE may be difficult, due to many possible abnormalities, with uncertain aspects and a need for highly specialized complementary tests [34].

Causes of reactive eosinophilia have a significant regional influence worldwide and regional large epidemiologic studies would be needed for a global evaluation of the problem. Despite the general perception that parasitic infections are the main cause of hypereosinophilia in tropical regions, recent studies found that an underlying malignancy was diagnosed with nearly equal frequency compared with infections [5]. Another important epidemiological aspect is the continuous increasing prevalence of allergic diseases in occidental and developed countries, with respiratory, drug and food allergies estimating to affect a large part of the population in the near future.

There is a clear need for harmonization of terminology and classification of eosinophilic disorders and for better quantification of HE-related end-organ damage. The proposal to implement a category of B symptoms for HE-related organ damage, similar to that already used in lymphoma stadialization, seems of practical value [10].

## Concluding remarks

Despite remarkable progress in understanding pathophysiologic mechanisms of eosinophilic disorders, there are still many unclear aspects, confusing terminology and gaps in diagnosing this very heterogeneous group of diseases. Beside an agreement upon clear and useful classification for disease phenotypes, there may also be an increasing need for disease endotyping and novel biomarkers, which may improve understanding of the disease pathophysiology. Classification based on disease endotypes may also have a direct impact on disease management and prognosis, considering personalized medicine. There is a clear need for specialized centers in eosinophilic disorders and for multidisciplinary teams, in order to implement international multicentric registries, updated diagnosis criteria and management guidelines. With the refining of therapeutic options and introducing of more targeted molecules, the prognosis of eosinophilic disorders might be significantly improved in the near future.

## Data Availability

Not applicable.
